# Prevalence and factors associated with metabolic syndrome among Tamils aged over 18 years in Jaffna district, Sri Lanka

**DOI:** 10.1186/s40200-015-0190-x

**Published:** 2015-07-22

**Authors:** Sivarathy Amarasinghe, Sandrasegarampillai Balakumar, Vasanthy Arasaratnam

**Affiliations:** Department of Biochemistry, Faculty of Medicine, University of Jaffna, Jaffna, Sri Lanka

**Keywords:** Fasting plasma glucose, High density lipoprotein, Hypertriacylglycerolaemia, Metabolic syndrome, Central obesity

## Abstract

**Background:**

The aim was to determine the prevalence and factors associated with Metabolic Syndrome (MS) among adults aged over 18 years in Jaffna district, Sri Lanka.

**Methods:**

This study was carried out as a community based cross sectional descriptive study in Jaffna district, Sri Lanka. Multistage stratified cluster sampling technique was employed. Total sample size was 544. An interviewer administrated questionnaire was used to gather data. Waist circumference (WC) and blood pressure (BP) measurements were recorded in standard method. Overnight fasting blood samples were obtained from all subjects. Fasting plasma glucose (FPG), high density lipoprotein (HDL), and triacylglycerols were analyzed by the enzymatic colorimetric assay (Semi Automated analyser Teco Diagnostics TC 3300). Modified National Cholesterol Education Program’s Adult Treatment Panel III (NCEP ATP III) criterion was used to define the MS.

**Results:**

Sample response rate was 95.3 %. Of them, 43.8 % (*n* = 224) was male. Prevalence of central obesity (WC for male ≥102 cm, female ≥88 cm) was 16.2 %. Prevalence of raised FPG (≥100 mg/dL), hypertriacylglycerolaemia (≥150 mg/dl), low level of HDL cholesterol (<40 mg/dL in males, <50 mg/dL in females) and raised BP (systolic BP ≥130 or diastolic BP ≥85 mmHg) were 23.9, 25, 79.3 and 36.6 % respectively. Prevalence of MS was 24.1 % (*n* = 123, 95 % CI: 20.4-28) and it was 26.8 and 21.9 % among males and females respectively. Participants living in urban area had higher prevalence of MS when compared with participants living in rural area (*P* = 0.015). Older age (*P* < 0.001) and smoking (*P* = 0.005) were risk factor for development of MS. Prevalence of MS among the participants with sedentary and active lifestyle was 29.9 and 23.7 % respectively.

**Conclusion:**

One out of four had MS. Old age, urban living and smoking carried higher risk for developing MS in Jaffna community.

## Background

MS is a combination of medical disorders that increase the risk of developing cardiovascular disease (CVD) and diabetes conditions such as raised FPG, abdominal obesity, high triacylglycerol, low high density lipoprotein and high blood pressure [1]. A recent study showed that the prevalence of MS ranged from 35.8 to 45.3 % in India [2] and 30.5 to 31.5 % in China [3]. A study carried out in urban Asian Indian adults using modified ATP III criteria, MS was present in 41.1 % [4]. Identifying participants with MS would help to prevent the increasing morbidity and mortality due to type 2 diabetes mellitus and CVD in Jaffna district. The aim of this study was to determine the prevalence and factors associated with MS among adults in Jaffna district, Sri Lanka.

## Methods

This was a community based cross sectional descriptive study among adults (above 18 years). A multistage stratified cluster sampling was used to obtain a sample that represents the adult population of the Jaffna district. Total calculated sample size was 544. The first stage of sampling the population in the Jaffna district was stratified into urban and rural sectors. The 32 clusters were allocated by probability proportion of size of the population in each sector. Considering proportion of the population, 7 and 25 clusters were selected from the urban and rural sectors respectively. Seventeen individuals were included in a cluster, so that the required sample size of 544 was scattered in 32 clusters. An updated list of households was available with the *Grama Niladari* officer. A household was randomly selected from this list in each of the cluster. In the field, the randomly selected house was located and visited as the first house. Next house to be visited was the one closest to the right side of the front door of the first house. This procedure was repeated until the required number of respondents was interviewed in each cluster. In every house visited, all eligible males and females above 18 years were listed in the summary form. The person to be interviewed was selected randomly using lottery method. This ensured the sex ratio of the study population.

Ethical clearance was obtained from the Ethical Review Committee, Faculty of Medicine, University of Jaffna. Written consent was obtained from individual participants.

Overnight fasting blood samples were obtained from all participants by experienced nurse. The relevant information such as demographic factors, socio- economic factors, family history, and other to ascertain risk factors and medical information was collected using an interviewer administrated questionnaire.

Age was categorized as 18–34, 35–49, 50–64 and ≥65 years. Physical activity level was classified into three categories viz. insufficiently active (no activity is reported /some activity is reported but not enough to meet categories of sufficiently active or highly active), sufficiently active (≥600 Metabolic Equivalent of Task (MET)-minutes/week) and highly active (≥3000 MET-minutes/ week) using the International physical activity questionnaire, which was translated into Tamil and pretested in the preliminary study.

Anthropometric measurements and blood pressure were measured in all participants by trained doctors. Body weight was measured with light cloths without shoes to the nearest 100 g using an electronic digital weighing scale. Height was measured using a stadiometer without shoes with the participant looking straight ahead. Waist circumference (WC) was taken by positioning the non elastic measuring tape midway between the lower rib margin and the iliac crest, at the end of a normal expiration. Hip circumference (HC) was taken with non elastic measuring tape as maximal circumstances at the buttocks. Blood pressure was measured in the seated position after the participants had rested for at least 5 min. The measurement was taken using the supported left arm at the heart level, using sphygmomanometer. Two recordings were taken and the mean was used for analysis. In the event of variation of over 20 mmHg between recordings, a third reading was done and the mean of the last two recordings was used.

FPG, HDL cholesterol, and triacylglycerols were analyzed by the enzymatic colorimetric assay (Semi Automated analyser Teco Diagnostics TC 3300). Data entry and statistical analysis were done using the SPSS Version 16 statistical package. The probability level was set as *P* < 0.05. Multivariable analysis, bivariate correlation and linear regression were used for analysis.

According to modified NCEP ATP III criteria, MS has five components such as central obesity, raised FPG, hypertriacylglycerolaemia, low HDL cholesterol level and raised BP [2]. If the participants possess any of the mentioned three abnormal parameters, they were identified as they are having MS.

## Results

Sample response rate was 95.3 %. Of them, 43.8 % (*n* = 224) was male. Prevalence of central obesity was 16.2 % according to modified NCEP ATP III criteria. Prevalence of raised FPG (≥100 mg/dL), hypertriacylglycerolemia (≥150 mg/dl), low level of HDL cholesterol (<40 mg/dL in males, <50 mg/dL in females) and raised BP (systolic BP ≥130 or diastolic BP ≥85 mmHg) were 23.9, 25, 79.3 and 36.6 % respectively (Table 1).

**Table 1:** Components of metabolic syndrome (MS) in the study population stratified by gender

Components of MS	Reference values	Male		Female		Total		*P* value
		(No)	(%)	(No)	(%)	(No)	(%)	
Central obesity	WC ≥ 102 cm in males, ≥88 cm in females	28	12.5	55	19.2	83	16.2	0.000^a^
Raised glucose level	FPG ≥100 mg/dL or previously disdiagnosed type 2 diabetes	60	26.8	62	21.6	122	23.9	0.176
Raised triacylglycerol level	TAG > 150 mg/dL or specific treatment of lipid abnormality	63	28.1	65	22.6	128	25.0	0.181
Low high density lipoprotein level	HDL < 40 mg/dL in males, <50 mg/dL in females	181	80.8	224	78	405	79.3	0.51
Raised blood pressure	Systolic BP ≥ 130 or Diastolic BP ≥ 85 mmHg or previously diagnosed hypertension	101	45.1	86	30.0	187	36.6	0.001^a^

Modified NCEP ATP III (2004) was used to identify the components of metabolic syndrome

^a^significance difference in components of metabolic syndrome between male and female participants

Prevalence of MS was 24.1 % (n=123, 95 % CI: 20.4–28) and it was 26.8 % (95 % CI: 21.1–33.1) among males and 21.9 % (95 % CI: 17.3–27.2) among females. Average values of anthropometric indicators, selected biochemical parameters and blood pressure of participants with metabolic syndrome were significantly higher when compared with participants without metabolic syndrome (*P* < 0.05; Table 2).

**Table 2:** Mean values of anthropometric indicators and selected biochemical parameters of participants with and without metabolic syndrome

Variables	Mean		
	Participants with MS	Participants without MS	*P* value
Weight (kg)	61.7 ± 13.2	55.0 ± 11.4	0.000^a^
BMI (kg/m^2^)	24.9 ± 4.7	22.5 ± 4.0	0.000^a^
WC (cm)	89.6 ± 12.3	79.4 ± 11.9	0.000^a^
HC (cm)	95.2 ± 14.3	88.9 ± 14.5	0.000^a^
WHR	0.9 ± 0.1	0.9 ± 0.1	0.000^a^
SBP (mm Hg)	129 ± 18	115 ± 16	0.000^a^
DBP (mm Hg)	83 ± 10	75 ± 11	0.000^a^
FPG (mg/dL)	126.2 ± 53.2	83.8 ± 24	0.000^a^
HDL (mg/dL)	32.8 ± 8.8	35.0 ± 9.9	0.025^a^
TAGs (mg/dL)	164 ± 38.3	92.6 ± 34.1	0.000^a^

Modified NCEP ATP III definition of the metabolic syndrome (2004) was used to identify the participants with metabolic syndrome

^a^significance difference between participants with metabolic syndrome and participants without metabolic syndrome

In the study population, gender was not significantly associated with MS (*P* = 0.212) (Table 3). Total participants were classified based on rural or urban living. In this study, 394 participants (77.1 %) were from the rural sector and the rest were from the urban sector. In rural area, 21.6 % (*n* = 85) of the participants had MS while 32.5 % (*n* = 38) of the participants had MS in urban area. Participants living in urban area had higher prevalence of MS when compared with participants living in rural area (*P* = 0.015). Females living in urban area had higher prevalence of MS than that of females in rural area (32.2 vs 19.3 %). Similar observation was also made among males (32.8 vs 24.7 %) (Table 3). Mean age of the participants with MS [54.1 (±12.2) years] was significantly higher when compared to the mean age of participants without MS [42.9 (±14.0) years, *P* < 0.001]. Among the age groups of 18–34, 35–49, 50–64 and above 65 years prevalence of MS was 9.0, 14.8, 36.6 and 50.9 % respectively. Same observation was done in both males and females. In the study population, old age was a risk for developing MS (*P* < 0.001) (Table 3).

**Table 3:** Prevalence of metabolic syndrome (MS) among the selected participants based on demographic factors

	Male				Female				Total				*P* value
	Participants		MS		Subject		MS		Participants		MS		
	(No)	(%)	(No)	(%)	(No)	(%)	(No)	(%)	(No)	(%)	(No)	(%)	
Gender	224	43.8	60	26.8	287	56.2	63	21.9	511		123	24.1	0.212
Sector													0.019^a^
Rural	166	77.1	41	24.7	228	79.4	44	19.3	394	77.1	85	21.6	
Urban	58	22.9	19	32.8	59	20.6	19	32.2	117	22.9	38	32.5	
Age (Years)													0.000^a^
18–34	48	26	5	104	85	29.6	7	8.2	133	26	12	9	
35–49	69	31.7	13	14.8	93	32.4	11	11.8	162	31.7	24	14.8	
50–64	75	31.5	28	36.6	86	30	31	36	161	31.5	59	36.6	
>65	32	10.8	14	50.9	23	8.0	14	60.9	55	10.8	28	50.9	

Modified NCEP ATP III definition of the metabolic syndrome (2004) was used to identify the participants with metabolic syndrome

^a^significance difference in components of metabolic syndrome between male and female participants

High prevalence of MS was found among professionals (41.7 %). Monthly household income was categorised as less than LKR 9000, LKR 14,000–19,999, LKR 20,000–31,999, and more than LKR 32,000. Prevalence of MS was calculated to each category. High prevalence of MS was found among subjects who received more than LKR 32,000 as monthly household income (33.3 %).

Among the smokers and the non smokers prevalence of MS was 36.8 % (95 % CI: 24.4–50.6) and 22.5 % (95 % CI: 18.7–26.6) respectively. Smoking was a risk factor for development of MS (*P* = 0.005), but type of smoking (*P* = 0.635) and frequency of smoking (*P* = 0.062) were not significantly associated with development of MS. Prevalence of MS was 32.8 % (95 % CI: 21.3–46) among the alcohol consumers and 22.9 % (95 % CI: 19.1–27.1) among the non-alcohol consumers. There was not statistically significant difference in prevalence of MS between alcohol consumers and non-alcohol consumers (*P* = 0.09). Physical activity level was categorized based on international physical activity questionnaire score. Prevalence of MS among the participants with sedentary and active lifestyle was 29.9 % (95 % CI: 20.5–40.6) and 23.7 % (95 % CI: 19.7–28.1) respectively.

## Discussion

In the rest of population in Sri Lanka, crude prevalence of MS was 27.1 % (95 % CI: 25.8–28.5), and age-adjusted prevalence was 24.3 % (95 % CI: 23.0–25.6). These values were similar to this finding. In the study population, there was no gender difference (26.8 % among males, 21.9 % among females). Prevalence in males and females were 18.4 and 28.3 % respectively [5]. In this study, 21.6 % of participants from rural area and 32.5 % of the participants from urban area had MS. Females living in the urban area had higher proportion of MS than those living in rural area (32.2 vs 19.3 %). Similar observation was also made among males (32.8 vs 24.7 %; Table 3). Katulanda et al. observed a similar distribution in other regions of the country. MS is more prevalent among urban dwellers in India too [6]. According to the present study, MS was more prevalent to be among the people who are more than 50 years. This is compatible with the findings of the previous study which was done in other parts of Sri Lanka [5]. Older age was significantly contributed to increased risk of MS [7]. With age, adipose deposition could be increased. It may lead to insulin resistance and higher LDL cholesterol, triglycerides, blood glucose, and blood pressure. Geriatric changes in the body systems may invariably contribute to this association.

High prevalence of MS was found among professionals (41.7 %). Sedentary lifestyle may be a major factor that contributes to MS in the professional category. Among Jaffna population high prevalence of MS was found among the participants who received more than LKR 32,000 as monthly household income (33.3 %). In other areas of the country, high levels of MS was observed in those with the high level of monthly household income [5].

Smoking was a risk factor for development of MS (*P* = 0.005). Several studies in the past have shown that nicotine leads to insulin resistance, has an antiestrogenic effect and increases the level of stress hormones like cortisol [8, 9]. In a previous study it has shown that risk of metabolic syndrome was stronger for active male smokers (pooled RR 1.34, 95 % CI: 1.20–1.50) than for former male smokers (pooled RR 1.19, 95 % CI: 1.00–1.42), and greater for heavy smokers (pooled RR 1.42, 95 % CI: 1.27–1.59) when compared with light smokers (pooled RR 1.10, 95 % CI: 0.90–1.35) [10]. There was not statistically significant difference in prevalence of MS between alcohol consumers and non-alcohol consumers (*P* = 0.09). Heavy alcohol consumption was associated with an increased risk of the metabolic syndrome by influencing its components [11]. In contrast to present study, in another study, males who never consumed alcohol had the highest prevalence of MS (12.3 %) [5].

Participants with sedentary life style had higher prevalence of MS 29.9 % (95 % CI: 20.5–40.6) than active (includes both moderately active and active) participants 23.7 % (95 % CI: 19.7–28.1). In Sri Lanka, prevalence of MS in the different physical activity categories of the IPAQ were; ‘inactive’−38.8 % (95 % CI 34.5–43.2), ‘moderately active’−33.5 % (95 % CI 30.9–36.1) and ‘active’−21.1 % (95 % CI 19.6–22.7) [5]. Sedentary life style compounded with the change in the nutritional pattern in Jaffna population makes them more vulnerable to non-communicable diseases. Regular exercise could increase energy expenditure and achieve weight loss and increase insulin sensitivity. Exercise could reduce blood pressure, increase HDL cholesterol and lower triglycerides levels.

## Conclusion

One out of four adults had MS. Old age, urban living, and smoking carried higher risk for developing MS in Jaffna community.
